# Dr. Alder's Proofs of His Theory from the Practice of Physic

**Published:** 1809-09

**Authors:** 


### Dr. ALDER's *Proofs of his Theory from the Practice of Physic.*

( In Continuation. )

IN aid of the demonstrations already given, I wish now to offer some *Proofs from the Practice of Physic.*

It must be understood that my subject does not demand of me to lay down any plan of my own for the treatment of fevers; and that (as I said before) it requires only that I specify how the modes of practice hitherto used agree with my indications.—My indications (as far as I can see at present) are

1st. To prevent an impeded passage through the lungs.

2dly. To obviate it when coming on, or formed.

3dly. To provide for or palliate its consequences.

And I apprehend that under these three indications may be arranged nearly the whole of the practice of physic.

I consider the practice in the first place as divided into two parts: the first relating to the prevention, and the second to the treatment of diseases.

*I profess,* 1st. to treat only of the prevention of fevers; but as the prevention of an impeded pulmonary transmission is the prevention of most diseases, it is impossible for me in some degree not to treat at the same time of the prevention of other diseases.

1st. *To prevent an impeded pulmonary transmission,* certain measures ought to be adopted suitable to every *particular* case: certain other measures proper for cases, as arranged under species, genera, orders, and classes. But the arrangement I would adopt is somewhat different from that found in Nosologies.

I must here, in a slight degree, allude to all the causes of an impeded pulmonary transmission, as care must always be taken to ascertain what these are in every case, and to remove or lessen them. But as I trust these causes are tolerably fully and clearly stated in my outlines, and as I wish to avoid as much as possible repetition, and the trouble of treating of diseases in general, I will be content with respect to a general specification of these causes, with referring to Part the Ist. of my Outlines. And I conceive it will be a considerable help to any gentleman who thinks it worth his while *fully to comprehend my meaning,* to attend to the ways in which I have shewn these causes act.—But it must not be expected that gentlemen can by a *transient glance,* make themselves masters of these causes and their modes of action, so as to derive much practical use; as, *in number*, they are so numerous, as to comprehend almost all the agents which act upon the human body; and *in their mode of action*, many of them so circuitous as well as intricate, that for a person to understand this well, he must come prepared with a correct and clear knowledge of the physiology and pathology of all the more important parts of the body; must know all the different susceptibilities, feelings, movements, and effects of the soul, and must besides constantly refer to the materia medica, and the discoveries in the meteorological and gaseous philosophy.

These causes of an impeded pulmonary transmission are divided into, 1st. Diseases, states and actions of the body; 2d. Passions and affections of the mind; and, 3d. External agents. And their mode of action will be found to be sometimes separately, sometimes in conjunction: sometimes upon the organs of respiration *directly*, (as distinguished into blood-vessels, air-vessels, muscles, cartilages, &c. and still minuter divisions,) and sometimes *indirectly*, and through the medium of the stomach or bowels, &c. or of each other.

But I intend to make a practical use of what I have there written, as far as my memory and other abilities will allow me, and is applicable to the present case.—Though at the same time I cannot but foresee that in doing this, according to my present plan, I shall be compelled to lay aside that systematic precision which I endeavoured to observe in my Outlines, and to take that manner and style which are more used in works of practice.

The articles used for the prevention of diseases, and particularly of fevers, are moderation in diet, mercury, bleeding, gentle purgatives, &c.; and on the other hand, the cold bath, bark and wine, a generous diet, and exercise, &c. also cheerfulness, and good air, &c.; and however contradictory many of these may seem, there are authentic testimonies that they all respectively have in different cases been used with success.

*Bleeding*, I am informed from a gentleman who practised in the West Indies, was performed upon a great number in one regiment there immediately after a campaign, and at the very time they changed a life of exertion for one of idleness by going into barracks; and those who were bled escaped free from fever, while most of the rest were soon seized with one which was very malignant, and many of them died.—Young, robust, plethoric people are very very apt to catch a fever in hot countries where the air is bad; while women, who have the periodical evacuation, those who have lived much on vegetables, as French people and negroes; and those who have just gone through a slight course of mercury, &c. are found to be much more secure; nay, even the weak and the aged oftener escape, that is, provided these last are free from all strong disposition to congestive diseases. The reason of this must be, that the plethoric *in such situations* are more liable to venous congestions. It does not uniformly happen that while such men are employed in an active life they escape; many are seized with fevers and other complaints in such places while on the most active duty: but when they change active to an inactive life, it certainly has happened that they have suffered most. They did so in this case, and Buonaparte's army did so in Syria.—But we are not, nevertheless, (I think) to conclude that it was *simply* the exercise which preserved them, any more than we are from what followed, that it was the rest altogether which made them ill. *Assuredly,* want of exercise *alone* is not one of the most powerful causes of fever; the Franks (as they are called) in eastern countries, during bad epidemic states of the air, seclude themselves; yet they, above all others, remain most secure. And as for the converse, we find that many, during laborious and even during common corporeal exertion, (whether they have been much and long accustomed to it or not, but more particularly if they have not) are in many instances seized with fever: and barracks are not always the healthiest places in the world; and they are not always fixed on the healthiest spot. However, there is in many kinds of exertion a peculiar pleasure, and something to infuse ardour into the mind; while there is, on the other hand, much inquietude among plethoric robust people under inactivity. Sailors wearied with disappointments, and garrisons besieged, and in despair, are very apt when the air is bad, to be attacked with fevers or the scurvy; while on the other side any thing which raises the mental ardour of the former, or gives sanguine hope to the latter, quickly brings their bodies into a state of convalescence almost inconceivable. Dr. Falconer in his Treatise on the Passions, and Dr. Alderson on the Factitious Metallic Tractors, detail many cases which go much farther than to support this.

But retracing my steps; bleeding these plethoric people in such cases unquestionably has done good, and has prevented fever! *How* it has done it is not difficult to see, it it would take off the superabundant blood and distension from the veins, and particularly from the lungs, and in doing this, much also of the præternatural heat and inquietude of the body; from which the excretories would be relaxed, and that perspiration allowed for a considerable time, which before was the effect of interesting exercise, and thus bring the state of these peoples' body somewhat nearer to the condition of that of those mentioned who often escape. And with respect to the condition of all these people, it must be perfectly clear, that when the blood is taken off by *menstrual evacuations*, when it is derived to the surface by a previous course of *mercury*, when it is allowed freely to escape from the surface by any inflammatory diathesis constricting the vessels of the surface, being prevented by living on *vegetable diet*, by *gentle purgatives*, or *mercury*, or even in many simple cases of *weakness*, or *advanced age*; or when this inflammatory diathesis is taken off by *bleeding*;—I say, it must be perfectly clear that in all these cases the blood will be much less inclined to congest inwardly, and much less apt to form great congestions there. And in addition, slight congestions, or indeed any congestions there formed in these latter cases, will have much less danger in them, and will occasion much less mischief.

The cold bath.—This, in warm climates, is too a preventive of diseases, and of fevers. It acts in several ways, 1st. it condenses the volume of the fluids, and abstracts heat: and, 2dly, while it takes off the heat of the skin and of the body, it by doing so, 1st. decreases the morbid irritability and inquietude of the whole body; and, 2d. it takes off that irritative diathesis before spoken off, as constricting the excretories. For perspiration may be checked by heat as well as by cold, and much oftener, and generally much more completely is so. But it is the heat of the body from an irritated state, and not external heat (further than as it causes this) which checks perspiration; and to prove the truth of this assertion, I need do no more than appeal to common observation. Those people who have a body properly prepared, have a free outlet by all the secretions and excretions, and can bear much external heat very well. While those who have somewhat of a turgid and irritated state of the vessels, will scarcely void any thing by the excretories, and are much incommoded or injured by heat. And when the corporeal heat is much greater, as in pneumonia, &c. and in fevers, the excretories are so constricted, that they will scarcely let off off any thing, though the blood be ever so violently driven to them. And in such of these cases as cold can be safely used in, the cold bath will relax the excretories and let off the sweat.—But, independent of these, the cold bath has another effect, it checks the motion of the blood in the veins, and prevents it from hurrying so fast up to the lungs. I have used it in two cases of great difficulty of breathing, accompanied with heat of the body, with a most decided advantage. Two stronger proofs, I think, could not present themselves than these of the efficacy of the cold bath in preventing the blood during a morbid heat of the body from hurrying up to the lungs; as one of these cases certainly (but I think both) were attended with a straightened thorax; and the difficulty of breathing was very great, and (except a little præternatural heat) the sole complaint.

I come now to articles apparently of a contrary description to almost all of the preceding; and which yet, nevertheless, have (according to the most respectable testimonies) frequently been used as preventives of some kinds of fever (or at least of fever in some situations) with much effect;—these are, BARK, WINE, AND A GENEROUS DIET. I know it has been recommended in several parts of the fenny districts of this kingdom, to use bark and wine, and a good diet, to prevent the autumnal fevers frequent in such places. And grog is much resorted to on ship-board. But these articles are not to be used except under certain circumstances; they appear to me to be adapted to a cold and damp atmosphere; as the former are for a hot one. But all of these are not so diametrically opposite in their nature as to preclude the propriety of using them *in some degree* in both. BARK we know is a medicine which *may be* given with good effect in a warm climate. And wine, with discretion, and a nourishing diet, often answer the same indications. They all, however, have better effect when alternated with articles of a somewhat different description, and particularly now and then with laxatives. It is necessary to have the stomach and intestinal canal in good order, and where these remedies will effect this, they may be of service. It is necessary also that the strength be not much sunk by *mere* debility; and to prevent this from happening, and to remove it, they are useful. But when the strength is sinking from *disease*, they may be much misapplied; as the remedies in such a case, it is clear, must be appropriated to the disease, and these in many cases may not be suited to it.

Under a cold atmosphere, it is much more necessary to take stimuli than it is under a hot or warm one: as nothing but these can, when a person is well, enable him to go about his occupations, or indeed to resist the influence of the cold. And among these a good diet certainly is the best. But as when the air is bad, the stomach may get out of order, a little bark may be requisite. A generous diet does not in a cold country expand the fluids, heat the body, and induce a morbid state of irritability in it as it does in a hot one; and of consequence, does not assist the action of bad air in the former as it does in the latter: but on the contrary, if the vascular action of the system be not kept up so as to resist cold, cold by constricting the skin, and by that means preventing the free flow of blood from the internal parts, may in some degree assist bad air. In an unhealthy season, great nicety is required in the admission and use of stimuli.

I come now to articles which have had more effect in the prevention of diseases and of fevers, and the contrary of which have had more influence in producing them, than any other whatever; I mean, CHEERFULNESS and GOOD AIR.

I have in this and other works, written much to shew the power of these agents and their contrary, and the extensiveness of their application; and I mean shortly to add something more. But whoever has been much conversant with practical writers, cannot fail to know they have much power and are much in action. How good air operates is evident enough, if gentlemen will but take the trouble of thinking about it. It gently stimulates the lungs, and makes them transmit their blood freely. *This is the only way in which it can operate*; but certainly if it do operate its beneficial and excessively benign effects *in this way, its operation is the strongest proof possible of the truth of my theory.*

There may be much more room for doubt about the mode of operation of *cheerfulness*, and its operation may too be more *complex*. But I have no room in this letter to enter upon the consideration of it. Dr. Falconer has paid a minute attention to it; so has Tissot.

( To be continued. )

*July* 10, 1809.

